# Perfectionistic Individuals' Understanding of How Painful Experiences Have Shaped Their Relationship to Others

**DOI:** 10.3389/fpsyg.2021.619018

**Published:** 2021-02-11

**Authors:** Vivian Woodfin, Aslak Hjeltnes, Per-Einar Binder

**Affiliations:** Department of Clinical Psychology, Faculty of Psychology, University of Bergen, Bergen, Norway

**Keywords:** perfectionism, perfectionistic, life story, narrative, qualitative, interpersonal relationships

## Abstract

**Background:** Perfectionism is increasing over time and associated with various mental health problems. Recent research indicates adverse childhood experiences may play a role in the development of perfectionism. In addition, perfectionism is marked by interpersonal problems with implications for treatment outcome.

**Aim:** This study aimed to fill an important gap in the predominantly quantitative literature field by exploring how individuals with perfectionism understand the relationship between painful experiences and how they relate to others.

**Method:** Nine individuals with perfectionism were interviewed using McAdam's life-story interview. Thematic analysis was used to analyze the interviews.

**Results:** Four themes emerged: “A childhood with big responsibilities,” “I am still the responsible one,” “Keeping others at a distance to protect the inner self,” and “Achieving physical distance to get a fresh start.” These themes are grouped into two overarching themes: “You can't always trust people” and “A distancing from others.”

**Conclusion:** Findings highlight taking responsibility and social distancing serve an important function for perfectionistic individuals in response to painful relational events. We discuss how themes of control and agency impact individuals' relationship to mental health and turning toward others for help. The findings provide greater complexity to understanding perfectionism as a “barrier to treatment.”

## Introduction

How do perfectionistic individuals experience the impact of painful life experiences in their relationships with other people? Perfectionism is considered a multidimensional personality trait, which consists of having high standards, self-criticism, fears and doubts about one's actions, and concern of other's evaluation (Frost et al., 1990). Perfectionism is therefore cognitive, value-driven, relational, and closely tied to the individual's sense of self-worth. Hence, the trait perfectionism may, for many, be strongly connected to their experience of who they are, both to themselves and in relationship to others. Early relational experiences facilitate healthy psychological development by learning that one's basic needs will be met. And as people grow, individuals' self-identity continues to be cocreated by one's greater context, in comparison to others and based on feedback one receives throughout life. Early relational patterns shape and form later patterns (Horney, 1945, 1950; Winnicott, 1960; Mikulincer et al., 2010). As a result, who we become, who we think we are, what story we tell about our life, and how we tell it are, to a large degree, shaped by the people around us and our relationships to them (McAdams, 2001). Evolutionary psychology theory suggests that by narrowing in on and learning from particularly negative experiences humans have adapted remained a part of the in-group and survived. However, negative events and stimuli receive in turn more attention (Baumeister et al., 2001). In response to particularly affect-laden events, individuals create scripts, which help them in predicting and similar events later in life, but also often build on overgeneralizations (Tomkins, 1991).

It is unclear why, but levels of perfectionism in our society have increased significantly in the last 30 years (Curran and Hill, 2019), at the same time as a growing body of research links perfectionism to a number of mental health problems, such as depression, anxiety, and eating disorders (Egan et al., 2011). There is a growing interest in understanding why and how perfectionism develops. Recent studies link perfectionism to adverse childhood experiences (ACEs), which include trauma, neglect, abuse, and family dysfunction (Chen et al., 2019). Two theories of perfectionism, the Perfectionism and Social Disconnection Model (Hewitt et al., 2017) and the Social Reaction Model of Perfectionism (Flett et al., 2002), posit that ACEs are an important factor in the development of perfectionism.

Perfectionism is a suggested transdiagnostic process in mental illness (Egan et al., 2011). In addition, perfectionism is theorized to be a barrier to treatment, and individuals with perfectionism may be less likely to seek help (Shannon et al., 2018). Individuals who score high on maladaptive perfectionism perceive less social support, hostility, and rejection and are less likely to experience relationship satisfaction (Habke and Flynn, 2002). Interpersonal difficulty appears to also affect patients with perfectionism in the therapy room, and less satisfying relationships among individuals with high pretreatment perfectionism predict poorer therapeutic outcome (Blatt and Zuroff, 2002). The negative relationship between perfectionism and treatment outcome is mediated by failure to develop stronger therapeutic alliances among perfectionistic patients (Zuroff et al., 2000). These findings may not be surprising as research especially highlights the importance of relational factors, called common factors, such as the therapeutic alliance, empathy, respect, and therapist's presence in psychological treatment (Wampold, 2013; Norcross and Lambert, 2018).

In order to improve access to and therapy outcome for this population, it is important to understand what individuals with perfectionism describe as difficulties in relating to others. The majority of research on perfectionism today is quantitative. As a result, the field can benefit from more qualitative studies, which can address how individuals who have perfectionistic tendencies experience and understand these processes. Some qualitative research has been conducted with individuals with perfectionism, which has highlighted marked interpersonal problems (Slaney and Ashby, 1996; Schuler, 2000; Rice et al., 2003; Merrell et al., 2011; Mackinnon et al., 2013; Farmer et al., 2017); however, to our knowledge, no study to date has focused primarily on interpersonal topics and how individuals make sense of important relational events in their life. In order to fill this important gap in the literature, this qualitative article will explore the following research question: How do highly perfectionistic individuals give meaning to the ways painful experiences shaped their relationships to others? We will convey the participants' own words to give meaning to their experience. Hence, this study aims at extracting experience-near themes of what the participants identify as important about emotionally challenging events in their life, their reflections about how these events have influenced their further life course, and how they conceptualize their relationships today.

## Materials and Methods

### Participants

We recruited nine individuals, two men and seven women, aged 20–50 years, studying at five different faculties at the University of Bergen. Participants were recruited from a large sample (*N* = 388) of students who signed up for a self-compassion course and scored in the top 5% on the Norwegian translation of the Frost Multidimensional Perfectionism Scale-Brief (Frost et al., 1990; Burgess et al., 2016; Woodfin et al., 2020). In total, 10 individuals were invited to be interviewed, and nine agreed to participate. The e-mail invitation stated that we were interested in understanding how perfectionistic individuals tell their life story. Participants had no prior relationship to the interviewer, the first author, but were informed that she would also be holding a course in self-compassion, which they had signed up for. Participants were not provided any incentives for participating in the interview; however, participants entered a lottery to win two movie tickets when filling out the survey questionnaire. The interviewer informed the participants that their responses were anonymous and confidential. Participants were informed that participation in the course was not contingent upon participating in the interview and that they could discontinue their participation from the study at any point prior to publication of the article.

### Procedure

All participants were interviewed in an office at the Faculty of Psychology at the University of Bergen. We used a semistructured life-story interview developed by McAdams (McAdams, 2008) which was translated to Norwegian. The aim of these interviews was to gain a greater understanding of perfectionism through the lived experiences and reflections of the participants. Interviews varied in length, between 120 and 360 min. McAdams life-story interview first prompts interviewees to give a brief 20-min synopsis of their life divided into chapters, emphasizing how they moved from one chapter of their life to another. Thereafter, participants are asked to identify key life events in each of the following categories: a high point, low point, turning point, high point in childhood, low point in childhood, main life challenge, primary health challenge, loss of an important person, biggest regret or failure, a spiritual moment, and moment of wisdom. Participants are then asked about their future projects, plans, dreams, hopes, and their beliefs on spirituality, politics, morals, ethics, and values. Finally, participants are asked to identify a single most important theme of their life. The interview is semistructured and suggested follow-up questions attempt to ask participants to identify a single scene with as much detail as possible: when and what happened, who was there, how did you feel, and what did you think. In addition, participants are prompted to reflect how they make sense of these scenes and what they think it says about them as a person or their life story.

### Data Analysis

The aim of these interviews was to gain a greater understanding of perfectionism through the lived experiences of the participants. The study used a hermeneutic–phenomenological exploration, group coding, and analysis with the use of NVivo 12 (QSR International Pty Ltd., 2020) to identify key themes among students with high perfectionistic tendencies. A reflexive hermeneutic–phenomenological approach is the interpretive and explorative analysis of the lived experiences of participants that highlights both the roles of the researchers and informants on the process and results. Because the approach acknowledges the researchers' impact on the work, it is essential to be aware and transparent about the unavoidable influence the researcher has to data collection, the analysis, and the interpretation of such work (Finlay, 2002; Binder et al., 2012). The use of qualitative methodology allows for a broad bottom-up exploration of perfectionism. As outlined by Braun and Clarke (2019), the first step in the analytic process consisted of familiarizing ourselves with the data. Each interview was reread multiple times, and initial impressions were noted and discussed. We generated initial codes for the data. The research question evolved as a part of the analytic process, originally from how individuals with perfectionism make meaning and cope with life challenges, to more specifically how participants described negative events affected them relationally. In our initial coding, we identified key relational themes. Narrowing the research questions allowed us to highlight the most common themes and reduce the scope of the article and report more in-depth findings. The data were recoded to address this particular research question. We searched for themes and studied the relationship between codes for overlap and differences through virtual representations. Themes were then reviewed. Not all themes were relevant to the research question and some were thrown out, whereas, others were further refined. Finally, themes were named and summarized. In reporting the findings, we aimed to tell the stories of participants by highlighting not only the similarities but also differences and variation. Because the interview highlights the individual's lived experience, it emphasizes the participants' own accounts, self-reflection, and narrative in order to increase our understanding of how they themselves make sense of painful events in their lives and the effect these have had on how they relate to others.

Finlay (2002) describes reflexivity as the immediate, continuing, dynamic, and subjective self-awareness, which can turn the researchers' inherent subjectivity from a problem to an opportunity from which meaning is cocreated. The use of transparency and reflexivity increases a qualitative study's trustworthiness. The process of reflexive analysis is a part of every stage of the research process from the preresearch phase to data analysis. In our work, we used reflexivity to discuss how our background, preconceptions, and subjective perspectives affected the research. Both supervision and notes on personal responses and reactions were used as springboards for conversation on our impact on and role in the research process. Because the first author has a different cultural background, Polish, American, and German, than the participants, Scandinavian, we discussed how cultural norms and expectations could affect the first author's analysis. For example, cross-cultural communication can differ in emotional expression and the degree to which one values individualistic goals (Triandis and Gelfand, 2012). In order to increase her understanding of cultural differences, as opposed to differences in perfectionistic tendencies, the first author also conducted six life-story interviews with individuals who had scored in the bottom 5% of perfectionism. These six interviews provided a valuable contrast to the phenomenon which we aimed to study, while normalizing culturally specific expressions and experiences. Ultimately, these interviews increased our understanding of the results of the thematic analysis by providing a contextual reference point for perfectionism within a group of students living in Norway.

### Researchers

The study was conducted within the Research Group for Clinical Psychology at the University of Bergen. The first author is a research fellow and psychologist with 6 years of clinical experience with training in self-compassion and mindfulness-based approaches. The second author is an associate professor in clinical psychology with 11 years of clinical experience and an interest in humanistic, experiential, existential, and relational approaches to psychotherapy. The third author is a professor in the Department of Clinical Psychology with 25 years of clinical experience with adults, adolescents, children, and families. His clinical approach is integrative, and he has training in mindfulness- and self-compassion approaches, emotion-focused therapy, and interpersonal/relational psychoanalytic therapy. All researchers have previous experience with qualitative research.

### Ethical Considerations

The study was approved by the Regional Committee for Medical and Health Research Ethics (Region North). All interviewees were given pseudonyms, and identifying information was changed to preserve anonymity.

## Findings

It is important to note that all but one informant disclosed having been bullied, abused, neglected, or experienced significant losses during childhood, but how participants described they reacted to these painful relational experienced varied. Our findings identified two overarching themes: “You can't always trust people” and “A distancing from others.” The first main theme, “You can't always trust people,” describes participants' experiences of not being able to trust other people and touches on aspects of personal control through responsibility and independence. The overarching theme “A distancing from others” describes participants' experiences of achieving both emotional and physical distance from other people who have or could hurt them (Table 1). Each main theme comprised two subthemes. “You can't always trust people” includes the subthemes “A childhood with big responsibility” and “I am still the responsible one.” These subthemes are divided by their timeframe. While “A childhood with big responsibility” outlines participants' history, the subtheme “I am still the responsible one” encompasses how this sense of personal responsibility still affects them today. The overarching theme, “A distancing from others,” is composed of “Keeping others at a distance to protect the inner self” and “Achieving physical distance to get a fresh start.” While “Keeping others at a distance to protect the inner self” illustrates how participants have achieved or try to achieve an emotional distance from other people, “Achieving physical distance to get a fresh start” outlines the physical distance achieved by, e.g., moving or switching schools. In this study, we describe frequency of the categories in participants' accounts, where “all” refers to all participants, “most” refers to all but one, “many” refers to more than half, and “some” refers to less than half.

**Table 1:** Overarching themes of responsibility and distancing.

**Overarching theme**	**You can't always trust people**	**A distancing from others**
Subthemes	A childhood with big responsibilities	Keeping others at a distance to protect the inner self
	I am still the responsible one	Achieving physical distance to get a fresh start

### You Can't Always Trust People

All informants described dramatic relational life events in childhood. Many described how other people had failed or hurt them. Informants highlighted the importance of taking matters into one's own hands and becoming the responsible one because one cannot rely on others. All informants discussed responsibly in various areas of their lives. For some, this was especially important in their youth and current relationships, whereas others highlighted maintaining control over personal qualities that could be judged by others, such as mental health, appearance, or performance.

#### A Childhood With Big Responsibilities

The first theme describes informants' experiences of having to fend for themselves and be the responsible one at a young age. All interviewed informants described painful relational experiences in childhood. These painful experiences varied in degree and nature. Some informants discovered that caretakers were unstable or unaccountable, some experienced painful losses, whereas others found that peers could be unfair and even malicious. Most informants narrated their story with a strong sense of personal responsibility, as if they could have done more to affect their circumstances, even as children. For some, taking responsibility and being independent early on did not feel like a choice but a survival strategy. Some informants explained that these painful experiences resulted in a feeling of losing their sense of safety, something that has remained with them.

Like many informants, Agnes experienced a lot of stress as a child. For Agnes, it took the shape of a “lump” in her chest that she carried with her, “all difficult events and all, all my fear, and everything was like a big compact mass, I didn't know what it was, but I knew it had always been there.”

Susie also recalled feeling a lot of responsibility early in life both at school and at home. She described how upon finding her dad unconscious at 12 years old; she was the only person in her family to attempt to save his life.

“Then I went upstairs, and then I saw my dad lying unconscious in bed. And my family was just standing there and like, just screaming at him from the sideline, and I jumped up on the bed and said we have to do CPR, he isn't breathing; we have to do something. That's when I started doing it…”

Dana describes feeling responsible for sexual abuse because it happened more than once: “I had a feeling, especially because it happened more than once, that there is something wrong with me, since I was chosen by two different men who have done this to me.”

Several informants described that taking responsibility as a child did not feel like a choice, but something they were forced to do so that their life would not fall apart. Agnes explained, “I was always really afraid, that if I didn't take responsibility, everything would fall apart.”

Like many informants, Thomas was also forced to be independent and fend for himself at a young age. He recalled how an angry stepmom inexplicably and without warning kicked him out as a teenager. The episode irreparably damaged his relationship with his parents and extended family.

“…she grabbed everything that was in my room and threw it out of my room (laughs). And that was hopeless and like… and incomprehensible, really. But what happened, and then I had to move in with my grandma. But what, what happened afterward, that was worse, really. Because then I lost contact with my dad, because she came up with stuff, things that weren't true, like… about me. That I… yeah, all sorts of things, that I hit her for example. That I smoked weed in my room and stuff. And all this embittered the whole family, so it wasn't just my dad, but aunt and uncles…”

Several informants highlighted how learning to fend for oneself went hand in hand with losing a sense of safety. Thomas recounted this when he described the upside and downside to being sent away to live at a hospital in the 60s when he was only 4 years old.

“The positive is that I became really independent at the time, like I had to manage alone. And, I think it has to do, I am good with people and stuff, I think it's so, I don't know if its connected, or if maybe it's something I just inherited, or something I just imagine maybe. At the same time as I believe that, really like, on the negative side, it really feels like uncertainty, yes, yes a lack of safety…”

Although Lilly described having a generally positive childhood, she shared the same feeling of fear at being left to fend for herself and a sense of losing safe boundaries upon finding her dad sleeping next to a beer bottle. Lilly, like other informants, explained the feeling has stayed with her ever since.

“The feeling I had then, it's really weird, it stuck with me. Exactly as if I saw him as an alcoholic, as if something had happened to him, as if he had died. But he had just renovated and had a beer. At least that's what mom says happened. So sometimes I wonder what that means [….] Mm. And what that says about me (laughs). I don't really know. But I've always been, I have always had a need for safe boundaries, and I didn't feel like I had those then. I felt a little as if… I had been left to fend for myself.”

#### I Am Still the Responsible One

While the first theme highlighted informants' experiences of having had a childhood with a lot of responsibility, the second theme reflects participants' experiences of taking a lot of responsibility today. Many informants underlined the importance of taking responsibility moving forward after learning that other people cannot always be trusted. This personal responsibility is described as extending to many areas, most notably mental health and relationships. Some explain that you can only hold yourself accountable for your own happiness because that is the only thing you can control. Informants exemplified personal responsibility in relationships as taking responsibility for how you present yourself in affecting others judgments of you, to not let others down and thereby avoid losing them, being more critical of other people so not be hurt and treating others well to in turn be treated well.

Several informants underlined the importance of personal responsibility in mental health. Lilly explained that you yourself have the most power to affect any situation, including your mental health: “And it's incredibly fascinating, mental health, how it comes and goes. But at the end of the day, it's really just we, ourselves, who have the most influence.”

Laila similarly discussed responsibility in mental health in terms of hard work. She summarized that everything is possible if you work hard. She explained you cannot blame your circumstances, only yourself.

“…those who work hard… anything is possible. So every now and then, it's like, if I get depressed, or can't do something because of anxiety, I think it's my own fault and doesn't actually have anything to do with illness.”

John was misdiagnosed with bipolar disorder, which had devastating consequences for his self-esteem, how he was later treated by health personnel, and his ability to trust. However, he explained that he primarily blamed himself for putting himself in that position: “I should have been more, I should have thought it through more before answering maybe, I should have presented things differently, or the *way* I talked was misinterpreted.”

He also described not being more critical and getting help in the first place as one of his biggest regrets: “…that I wasn't more critical and, that I in a way, *allowed* myself, to seek help, that I didn't manage to normalize things for myself, and just accepted the answers I got and just, wasn't more critical.”

Similarly to John, Dana described how important it is for her to also take responsibility for how others see her: “…there is so much that I can't control but there are things I can control: how good I am at a job, and how people see me. Which attributes people *see* that I have.”

For Dana, taking responsibility was important to keep the relationships she had left after she lost both her parents at a young age. Out of fear of also losing her older brother, her early 20s revolved around taking responsibility for maintaining their inheritance, their family home. She described that because she was terrified of losing her brother, she became terrified of losing the house: “…I was the one the responsibility fell upon. So I was scared to death that I wouldn't be able to do it. I thought, I can't lose the house, I have to keep the house. Now everything is on my shoulders, I have to perform.”

Thomas expanded and summarized this as: “You are the architect of your own fortune.” And explained taking personal responsibility also extends to how you treat others because this in turn comes back to affect you: “You yourself are really important as a person, the protagonist in your own life, and if you care about those around you, you can also influence them, and that in turn can affect you.”

### A Distancing From Others

In “A distancing from others,” informants describe how they have established distance, both physically and emotionally, from other people in order to protect themselves. By keeping other people at arm's length, they protect their inner self from re-experiencing relational pain. However, participants also describe that emotional and physical distance comes at a cost and describe how insulating oneself from intimacy can also hurt.

#### Keeping Others at a Distance to Protect the Inner Self

Many informants described learning that people can be unreliable, unstable, untrustworthy, and sometimes dangerous. These experiences taught many participants to emotionally distance themselves to keep people out, end relationships, or otherwise insulate their inner self from potential harm. In contrast to the second theme, “I am still the responsible one,” in this third theme participants do acknowledge the power other people can have, but they outline their attempts to minimize the effect this can have on them. In this theme, participants share the various ways they achieve emotional distance from others in order to protect themselves. For some informants, it is difficult to make room for themselves in relationships out of fear of losing those important to them. A couple of informants described the difficulty in distinguishing their own needs from those of others. Many participants discussed the importance of boundaries in their relationships, in order to stay safe and make room for themselves: their needs, wants, and desires. Overarching for this theme is the shared experience, yet different ways in which informants keep others at a distance and protect themselves by not fully revealing who they are or what they need. By sequestering their inner self and creating emotional boundaries, some describe not only experiencing isolation, but also a sense of protection from being hurt, being taken advantage of, or losing oneself.

Boundaries of walking away were important for several informants for the sake of staying safe. Agnes described wishing she had had the knowledge and power to walk away when she was experiencing emotional and physical abuse as a child.

“I think that it may be defined my self-image for a long time. But yeah, most of all I remember, I remember exactly what happened. And I had a flashback memory of everything. I remember I was so, like I was so scared, my entire body was like, yeah. Really tense and I felt like I was forced to receive the comment and just endure it because I had no sense of walking away.”

For Laila, friendships in themselves felt unsafe because she learned her friends could move away and disappear, and she recalled the moment she realized she would rather have no friends than experience one more loss: “I have problems trusting people, I expect that everyone will disappear and stuff, and often that scene comes back then. That's when I realized that I had to quit, that friendship and stuff, friends disappear, no good things. Just… So there was a lot of sadness and frustration and a lot of fear.”

Lilly described how she feels judged all the time, even by her doctor, so she tries to stay away.

“…I think that everyone is thinking something about me. And especially my doctor. If I time after time go on sick leave, that doctor thinks “what an idiot, can't you handle more than that[….] And then, then it's better to stay away, then you avoid that.”

For Dana, it became important to not trust people, because she learned early on that her vulnerability could be used against her to bully her: “…it wasn't safe to be myself around people because you never knew what they would use against me or use to hurt me.”

Dana described herself as alike the cats that she fosters, who do not trust people anymore because of the pain they have experienced: “I really see myself in these cats too, like scared, and hurt by something, don't trust people.”

Dana described that there has been too much risk involved in letting people in. In addition to being able to hurt you or disappear, they might discover you are not good enough and reject you.

“…first and foremost I am afraid to open myself to others, to tie myself to others to stay, to be hurt again, or that I will lose people *again*. Mmm. Or that I'm not good enough, or if I'm not happy or smiling or positive, and the kind of person that people would like, that maybe people won't like me or want to be with me. That I always, that I always push people away has always been my fear, scared of, I have always tried to make myself strategies to not be hurt in any way.”

Romantically she described how she has not allowed anyone to hurt her, because she has isolated herself or has broken off relationships before they got too serious, “…I think I have never, never opened myself up to be hurt.”

Laila explained that her dad's inconsistent comings and goings made her question whether she was loved as a child. Like Dana, she found strategies to lessen the pain. Laila gave up hope: “the way I handle it now, I just stopped hoping really, I have stopped thinking he will change, that he will suddenly be ready to be a dad.”

Katie described that it is difficult for others to read her and how she is feeling. She says she is protecting herself because she does not really trust her friends will stay her friends:

“It's a bit like, a defense mechanism that I have with friends and stuff because I think like, I always have it in my back of my mind that they don't really want me, and so I never manage to really trust them. And that's why I don't want to tell them or I don't want them to know things, in case they move on or don't want to be friends anymore. I don't want them to know things about me. I don't totally trust them.”

A few informants described that not setting clearer boundaries and expectations was one of their biggest regrets because their needs were not met or prioritized when they should have been. Laila described, “I spent a lot of time regretting that I haven't been clearer with people about what I need, or what I deserve. That I could have been a little more demanding in a way. I regret that I kept going to that terrible therapist, because I knew it didn't really work.”

Setting clearer boundaries to prioritize her own needs was also an important subject for Dana. When recounting her experiences, Dana took a lot of personal responsibility for not doing more to protect herself from abuse and unwanted sexual advances. She explained the spiral of how setting others' needs before her own resulted in her experiencing even lower self-worth and that her lack of boundaries affected her experienced value as a person.

“I'm not very good at setting boundaries, that's made it difficult for me to set boundaries, and that other people's needs have come before mine has then affected me by giving me really low self-esteem and self-image. When I've always ignored my own needs and my own boundaries [….] I didn't know what was normal in a way, between two people. I didn't feel like I could say no to certain people, and I just had to do the things I didn't want to do. And I couldn't set boundaries as I got older…. So it made me feel like I, yeah, like, that it was at the expense of my own worth, my self-worth as a person.”

Thomas recalled that being isolated from most of his family due to his stepmom's lies made him more dependent on romantic relationship: “It's connected to, cling to a relationship, a relationship because I need safety.”

Thomas explained how this dependence on his partner made it difficult to prioritize his own thoughts and desires. Always wondering what she was thinking or wanted him to do would overshadow his own needs.

“I became really… controlled, by what she thought and felt. And that then affected us on and on and on and in the end, right, it was like we were dependent on, another person that had to, ‘What did *you* think?' Not like, what do I think and what do I feel like, but ‘what would *you* like for me to do?' It was a bit like that in the end.”

Some informants also recounted discovering that boundaries are important as someone who wants to help others. John described learning through his parents' separation and mental health problems that it is possible to take too much responsibility for others. He described how he, as a child, felt he played a very important role in his parent's separation and his mom's hospitalization and how this pattern has followed him into other relationships.

“I assume a lot of, what shall I say, responsibility for wondering about other's situation. Or before, in a way, more than, more than a kid should, thinking, and being like ‘mom isn't doing so well' and done with that. Not like, not like I should, as if I have some sort of central role in all of that. I don't, I didn't have one. I, I just take a lot, a lot of responsibility for other people's feelings. Like the two relationships I was in. The two break-ups, and friendships. I assume a lot of responsibility for feelings, it's not necessary.”

He also described the importance of figuring out when you have given enough of yourself and setting boundaries in relation to people who are struggling.

“I feel like you have to in a way, set boundaries for how much you want to give of yourself to others. You can't carry other people's feelings, you can't. And I can't control or take responsibility for everything that happens with those around me. It's important to limit a little bit. To be selective of time.”

Dana described how constantly learning to adapt to new people and new places when she moved made her a chameleon that lost touch with what she needed.

“I called myself a chameleon for many years, because I felt like I was really good at fitting in in new settings, but in the end it was like I couldn't… I was controlled by the outside settings more than finding out who I actually was and what I wanted.”

For Paula, feeling like she was finally acting on her own accord and following her gut has been one of the most powerful moments of her life.

“It sounds a bit tragic, that it's one of the points that sticks out, out of all of them, like all, when I've won the lottery or, but…. I think, what it says about me, if you go in depth, it says that it's important to me to make decisions in my own way, in my own time.”

Laila also described trying to strike a balance between her own needs and fulfilling expectations in order to make more room for what she needs: “So it has to do with, it has to do with finding a balance between taking care of oneself and at the same time like fulfilling expectations from others and myself.”

Laila summarized the feeling of emotional distance from others: “I definitely have a consistent feeling of the world around me fitting together and people around me feeling community and at one with nature with meaning and purpose, and I instead exist on the side for myself.”

#### Achieving Physical Distance to Get a Fresh Start

The final theme describes how informants achieved distance from difficult situations or relationships by moving on physically. Many informants described untenable situations that they could not remain in any longer and the need to achieve a fresh start through distance. They described being unable to tolerate a given situation out of loneliness, fear, pain, or being fed up. Many informants described that it is important, not only to cut emotional ties to establish distance, but at times also the necessity of getting away physically. They described moving, in several cases abroad, as an attempt of starting anew and leaving painful experiences and hardships behind. For some, this felt like a big turning point in their lives. However, there was a lot of variation in the extent to which participants experienced moving as successful. Several of the informants reflected that it is impossible to truly get away because the pain always catches up with you. Other informants point out that you can never truly shake the experience of not being good enough even if you change your life. However, some described successfully achieving physical distance or a sense of belonging abroad, but lament that they had to leave the distance behind and come back home to old struggles. A couple of informants planned on moving abroad again in order to start a new life.

Many informants described experiencing positive changes upon moving. Susie was able to see her home in a new light when she returned from travels abroad.

“I saw everything in a new light because I had lived in a really big place where I had seen; we had visited big cities abroad, and I had seen that the world is so much bigger than I had imagined. So before I thought that Bergen was a big city, but then I came back and I realized that Bergen is a really tiny city (laughs) [….] And it was like it didn't hit me before I got back and even when I…. I thought it was a pretty big city, it's just itty bitty, I don't understand that I never realized that this is a tiny valley with lots of houses (laughs).”

Katie also described moving several times to get away from home because it was frustrating and lonely to be the only person who cared about school.

“Where I come from is a tiny village, where almost nobody cares about school, not parents. Almost nobody has an education, so there's a lot of people who never finished high school. They don't care at all. I got so frustrated. This is important! (laughs) And nobody hears, nobody cares about school, nobody reads, nobody shows up to class, they don't show up, so that was a role I tried to push. […] It was frustrating. That's when I applied to go to high school in a different city.”

She also described later moving abroad as a great experience, where she finally found likeminded friends and felt she belonged. For her, it was painful to have to come back.

“…it was a very turbulent school year, where I felt I didn't want to be in Norway any longer. I wasn't thriving there, it was nice abroad. So I really wanted to study abroad again also after high school. But then… it costs a couple million a year, the schools I want to go to, they don't offer stipends, you get extra stipends for those schools, but if you want to go to a top university it's how much of a stipend you get and how much extra stipend you get abroad. So I would've economically destroyed [my parents] if I had done it.”

Laila described all the challenges she experienced when moving to live on her own for the first time. Nevertheless, she felt satisfied and independent despite all the small irritations. For her, living by herself represented her own health and being normal, like others.

“I remember the internet didn't work immediately, and I remember I was struggling to screw on a leg on an Ikea bed, and at the same time I was really like… content, felt that I was normal, it made sense that this is what I should be doing. So even those things that were irritating, like the internet not working, but now I am actually in a situation in my own studio apartment where the internet doesn't work… that's something all students experience, that is how it's supposed to be…. I don't really know what it says about me. But maybe that I really have a need to function as well as others, that has been really important to me.”

For John, it was also important to move out when he began high school because he had lost hope. Later moving out to join the military was a key turning point for John, where he could redefine himself and experience mastery. But he also reflected that despite this he did not manage to totally shake his insecurities: “…that's when I started doing really well in a way, but that self-consciousness stuck with me.”

Dana described having moved abroad many times and how she tried to escape pain, which ultimately did not work.

“…my move was more based on, if I move to a different country I can escape all the painful feelings and in a way, reinvent myself, ehm, without that being a problem. But I realized quickly that it doesn't work like that, so I moved back home again with mom.”

At the same time, Dana had plans to move abroad again soon to start a new life with a new partner.

“I'm going to move abroad to be with him, and establish myself there, and get a job and yeah. That's going to be the next chapter. Ehm, moving back abroad, and beginning, like continuing my life there. I feel like my life the last 15–20 years has consisted of one day at a time and not seeing any future. And being really like, locked inside, by all of these feelings and not getting anywhere, and now I feel like I'm beginning to break out! […] And I actually *have* to start the rest of my life.”

Agnes similarly moved abroad in order to get away from difficulties back home.

“I had decided really early on that as soon as I could, I would get away from it all, from the city, from my family, and everything that had been. So I just moved abroad alone. And that was really important to me, I know it was really important for me to get distance to it all, and understand that I can be a person without all the things that were difficult for me. Without being stuck in that life pattern.”

But Agnes also described that the past always ends up catching up to you.

“I've understood now that I wasn't done with all the old stuff. There was so many things that I handled then and there, and that I got help with then and there, but I never got help understanding what that had done to me as a person.”

## Discussion

In discussing the findings, it is important to note that relational trauma in childhood played a large part in participants' understanding of how they related to others today. Winnicott's theory of development presents how the interaction between the environment and the child shapes an individual's mode of being, true and false self and authenticity (Winnicott, 1960). The true self is used by Winnicott to describe an authentic, spontaneous, sense of “feeling real” self, which is contrasted with the false self, which masks the true self to protect it. Although Winnicott (1960) also emphasizes the child's role in this interaction, he proposes that failures of both omission and commission of the parent cause reactions in a child, which can impede his or her ability to integrate his or her self. The participants in this study could often identify single significant moments when they lost a sense of trust or safety in their relationship to other people. Baumeister et al. (2001) describe that for evolutionary survival reasons, negative events have more valence and a larger impact on individuals than positive events. McAdams, the developer of the life-story interview, further divides how we tell these stories of hardships into two categories: redemptive or contaminated (32b). While redemptive stories highlight overcoming or the gains attained through adversary, contamination stories describe how negative events negatively impacted a previously good narrative (McAdams et al., 2001). In his affect-regulation theory, Tomkins (1991) further proposed that individuals create scripts in response to such affect-laden events. By comparing one affect-laden event with another affect-laden event, individuals attempt to predict and respond to a set of events, which may become a pattern of responding. For example, several informants generalized how a sense of a loss of safety, distrust, or hopelessness followed them into future relationships or was something they carried with them thereafter and could even be experienced bodily as a constant lump of fear. Horney (1945, 1950) describes that individuals can move toward, move against, and move away from others in response to environmental factors that produced experiences of hopelessness or isolation in a child. However, these modes of reacting are not pathological in themselves, but similarly to Tomkins (1991), she suggests that it is rather the inability to vary one's response, which can become problematic. Horney hypothesized that a single mode of reacting could become a neurotic trend, a character trait in an individual, and proposed three types: compliant, aggressive, or detached.

### The Pride and Pain of a Childhood With Big Responsibilities

In the first theme, participants described with in part pride and part resentment how they became the responsible and independent individuals they are today. The informants describe themselves as adaptable survivors who adopted these traits in order to cope with difficult life events or traumas. However, there is great nuance to the survivor story, and not all stories are redemption stories that highlight gains. Some individuals also emphasize that becoming independent did not feel like a choice but rather something that was forced upon them. While Baumeister and colleagues' theory of how bad events have stronger salience than good normalizes this phenomenon, McAdams et al. (2001) report that contamination stories, stories that go from good to bad, correlate with mental health problems such as depression. Baumeister et al. (2001) also propose that experiencing multiple negative events may have a snowball effect. As one informant explains, because she was the victim of abuse more than once, it became more difficult to externalize these events and thus blamed herself. Cumulative negative relational events may make it more difficult for an individual to ascribe that a negative event happened by chance and does not have bearing on their identity or self-perception. Tomkins (1991) describes in his theory of affect regulation that especially the type of events that elicit fear and shame, an emotion that attacks the self, can result in compliance of what Winnicott (1960) describes as the false self and damages a child's ability to develop an integrated identity. Participants explain that they may have gained a sense of control after adverse experiences, but that control was not awarded to them by their own choosing. Tomkins (1991) elaborates that similar events that mobilize these effects of shame and fear and interfere with interest and enjoyment will result in conflicting feelings of hope and apprehension of redisappointment. Participants describe attaining their sense of agency with similar ambivalence. This sense of personal control and responsibility is described as a byproduct of pain, a coping strategy developed by the environmental factors that produced them, at the same time as it is revered by informants as their metaphorical life raft. In particular, participants identified gaining a sense of independence while losing a sense of trust in others, safety in relationships and safe boundaries. Having a sense of being left to fend for oneself at a young age touches upon experiences of abandonment and grief of the loss of the freedom and carelessness that often accompanies childhood, but what they may feel was stripped from them. Horney (1945) describes the detached type as individuals who lean toward moving away from people and are neither interested in fighting nor belonging. This closely resembles the fearful-avoidant attachment style in adults who identify with being uncomfortable getting close to others, difficulty trusting and depending on others despite wanting emotionally close relationships, and worrying about being hurt by others if one gets too close (Bartholomew and Horowitz, 1991). Horney (1945) describes that these individuals often have a strong need for self-sufficiency. Several informants underline that there was a single significant moment when it felt as if these boundaries, this safety, or the floor from under them was lost. The feeling that arose is described as a sense of being all alone, the only person who will watch out for themselves giving rise to the fierce self-reliance, independence, and anxiety that is central in how we understand perfectionism today.

### I Am *the* Responsible One

In the second theme, participants conveyed taking responsibility in relationships as an important value to them today. As Tomkins (1991) discussed, individuals create scripts in response to such affect-laden events, which aid them in creating a pattern of predicting and responding to similar events. Even though the independence, responsibility, and self-sufficiency are described as a byproduct of pain, it is also described as an important trait among participants. Informants detail how hard work gives them the power to affect any given situation and most notably in how they are evaluated or judged by others. This strong sense of personal control and agency is also related to mental health. However, this again comes at a cost because when unable to control emotions or mental health, informants describe that they only have themselves to blame. Tomkins (1991) describes the basics of affect motivation as maximizing positive affects, minimizing the cause of negative affects, and minimizing affect inhibition. However, informants detail not only how they aim to minimize the cause of negative emotions, but in contrast with Tomkins (1991), also how to inhibit their negative affects in order to control how they are viewed. This implies that expressions of mental illness may be considered a personal weakness that has not been successfully controlled. A few informants also describe their emotions with contempt, as if a personal betrayal to their sense of control. This contempt of the true self, as discussed by Winnicott (1960), is the result of shame or fear. Tomkins (1991) suggests that compliance, the concealing of the true self in exchange for compliant behavior, serves to minimize these unwanted fear and shame-laden emotions. Some informants also describe, with regret, situations in which they turned toward others for help. By lamenting that they did not manage to help themselves and regretting they conveyed themselves poorly, they again highlight their strong beliefs and values of self-reliance, hard work, and independence. Pairing this with sole personal fault for allowing oneself to be judged also effectively maintains a sense of personal control but strips others of perceived agency and will, because what lies implicitly in taking all responsibility is that others have none or less. The implications of resilience through perceived control and inaccurate liability may pave way for the extreme self-criticism that accompanies the inevitable, unpredictable, and uncontrollable situations all individuals face in life.

### Protecting the Inner Self From Double-Edged Others

Emotional distancing can serve as important coping and survival mechanisms by which one can protect oneself from being hurt, rejected, or overwhelmed. As Baumeister et al. (2001) explain, a single negative event in relationships is more powerful than any positive event, naturally giving more weight to avoiding negative relational experiences. If one learns that the risk of relational trauma is more dangerous than the pay-off of relational connection, perfectionism may have an important adaptive role in avoiding further decreases in well-being and increases in negative affect. Horney (1945, 1950) describes that individuals who move away from others, also called the detached type, distance themselves from others to consciously or unconsciously avoid emotional involvement. For the informants, emotional distancing may have helped them endure relational traumas by impeding the intimacy that can allow someone to come close enough to inflict more relational pain. However, emotional distance also comes with several disadvantages. Many informants also reflected that while they successfully achieved distance, they felt loneliness or fear around other people. By gaining this kind of relational control, one must sacrifice a sense of belonging, and Horney (1945); (Horney, 1950) explains that the detached type commonly experiences estrangement. As one participant recounts, she feels like the world around her is in community while she is watching it as an outsider. Through distancing, one may in one sense maintain a greater experience of control, but it may invariably contribute to increasing one's vulnerability to pain caused by others through isolation. Several informants noted that they adopt the way they present themselves in order to affect people's judgment of them and thereby spare themselves from disapproval or rejection. This closely resembles the hypothesis of Mackinnon et al. (2013), who in a large longitudinal mixed-methods study found that perfectionism positively correlated with themes of agency, yet surprisingly did not correlate with domains of communion, such as friendship, support, togetherness, and mutual dialogue. They explain these results by discussing that individuals with perfectionism may want to have close interpersonal relationships but nevertheless fail to for a variety of reasons (Mackinnon et al., 2013). By trying to maintain perfect outward appearances, individuals may attempt to adapt better to different situations. Nevertheless, as many informants recount, by prioritizing the needs and desires of only those around them to, for example, avoid critical evaluation, they also cede their own wants, needs, and desires. Horney (1945, 1950) describes that detached individuals may become numb to their own experiences, feelings, needs, and desires. Some interviewed informants similarly describe a loss of a sense of self. It becomes more and more difficult to differentiate what they want from what they believe others want from them. In other words, participants may to a larger degree view the world around and themselves through the lens of their perception of the judgment of others.

### Less Responsibility and More Hope in Moving Away

Similar to the previous theme, in the fourth theme, “Achieving physical distance to get a fresh start,” informants do acknowledge that life can at times be unfair, despite hard work and control. Almost all the interviewed individuals had lived abroad or moved away to start a new life or escape situations that they felt were intolerable. These themes resemble each other because both reflect detached types' tendency to “turning away” from difficulty (Horney, 1945). By moving and distancing themselves physically, they again exerted agency and independence by turning away from difficult situations. However, in contrast to the previous theme, by moving physically, they exhibit a stronger sense of boundaries and self-empowerment. The action of changing environment outlines a shift in blame. This implies that moving potentially reflects greater externalization instead of internalization of problems because the fault is in others or the situation outside of oneself. This is interesting because it also represents the release of the perception control and responsibility in any given situation, and thereby a new script of response to an affect-laden event. However, it is also paradoxical, because moving can represent both giving up on belonging and gives rise to hope for a new situation or context. However, findings indicate that this had varied success for participants. Some describe that the problems primarily lie within them and therefore were inescapable or caught up with them, whereas others felt freedom and found a sense of belonging elsewhere. This motivation to belong is what differentiates Horney's description of the detached type who is not interested in belonging, and the fearful-avoidant type as described by Bartholomew and Horowitz (1991). This implies that emotional and physical distancing may serve a protective function but not accurately portray an individual's motivation for emotionally close relationships. Moving ultimately had no unifying result for all participants, but rather affected the individuals in different ways. It is also interesting to note that a few informants expressed they hoped they themselves would change or had successfully changed by moving, again reflecting the ambiguous boundaries felt between their sense of self and the world around them.

### Limitations

There are several limitations to this study. First, although we investigated the relationship between adverse relational experiences and their effects on how individuals with perfectionism said they related to others, one cannot draw causational inferences about this relationship. The primary aim was simply to understand how individuals with perfectionism experience this relationship, and it may not relate exclusively to individuals with perfectionism. This study has a small sample and is based on in-depth interviews with nine student participants living in Norway, therefore limiting generalizability. Although we are concerned with reaching thematic saturation in qualitative methods, this does not preclude that more relevant themes or greater variation in themes could have become apparent with a larger or different sample. Finally, qualitative thematic analysis is a product of the informants, the interviewer, the context, the tools used, and the researcher's analysis. As a result, although the findings aim to stay as true as possible to the first-person experience of the interviewee, they will unavoidably be influenced by the relationship of all of the above.

## Conclusion

In this qualitative analysis of how individuals with perfectionism give meaning to negative relational events, we identified four main themes. These themes highlight the importance of responsibility and distancing from others in response to relational pain and trauma. These findings indicate that distancing may serve an important function for individuals with perfectionism. The themes lend support to previous perfectionism theory, which indicates that although perfectionism may correlate with deficits in topics of communion, such as interpersonal problems, this should not be confused with communion motivation (Mackinnon et al., 2013). Although previous research has identified that ACEs may play a role in the development of perfectionism, this article is the first that we are aware of to understand this relationship from a first-person point of view. These findings are also important in nuancing our understanding of the phenomenon perfectionism and its known mental health correlates. We discuss how themes of control and agency may impact individuals' relationship to mental health and turning toward others for help. Previous research identifies perfectionism as a potential “barrier to treatment.” These findings allow for greater complexity in our understanding of the relational mechanisms that may contribute to avoiding seeking help, resistance to treatment, and reduced treatment outcome in this population. Participants described a strong sense of personal responsibility and fear of being hurt. This has important clinical implications for building a strong therapeutic alliance. In addition, these findings nuance our understanding of individuals' ambivalent motivation for emotional distancing and do not necessarily imply a lack of desire for intimacy. Difficulty with emotional closeness and a strong sense of personal control should also not be misjudged as they are described as survival techniques that have at times played an important role for participants. However, findings should not be used for generalizations of individuals with perfectionism, but rather shed light on the complexity of these relational mechanisms. Future research may want to further explore how and when individuals with perfectionism feel safer in relationships and clinical settings.

## Data Availability Statement

The datasets presented in this article are not readily available because Datasets generated for this study are available on request to the corresponding author. Requests to access the datasets should be directed to vivian.woodfin@uib.no.

## Ethics Statement

The studies involving human participants were reviewed and approved by the Regional Committee for Medical and Health Research Ethics (Region North). The patients/participants provided their written informed consent to participate in this study.

## Author Contributions

VW and P-EB contributed to the conception and design of the study. VW conducted the data collection and interviews. VW, P-EB, and AH contributed to the thematic analyses. VW wrote the first draft of the manuscript. P-EB and AH wrote sections of the manuscript. All authors contributed to manuscript revision, read and approved the submitted version.

## Conflict of Interest

The authors declare that the research was conducted in the absence of any commercial or financial relationships that could be construed as a potential conflict of interest.
